# Pharmacological Inhibition of NOX4 Improves Mitochondrial Function and Survival in Human Beta-Cells

**DOI:** 10.3390/biomedicines9121865

**Published:** 2021-12-08

**Authors:** Andris Elksnis, Jing Cen, Per Wikström, Per-Ola Carlsson, Nils Welsh

**Affiliations:** 1 Science for Life Laboratory, Department of Medical Cell Biology, Uppsala University, SE-751 23 Uppsala, Sweden; andris.elksnis@mcb.uu.se (A.E.); jing.cen@mcb.uu.se (J.C.); Per-Ola.Carlsson@mcb.uu.se (P.-O.C.); 2 Glucox Biotech AB, Frälsegårdsvägen 8, SE-179 97 Färentuna, Sweden; per.wikstrom@glucoxbiotech.com; 3 Department of Medical Sciences, Uppsala University, SE-751 85 Uppsala, Sweden

**Keywords:** NOX4, beta-cell, mitochondria, cell death

## Abstract

Previous studies have reported beneficial effects of NADPH oxidase 4 (NOX4) inhibition on beta-cell survival in vitro and in vivo. The mechanisms by which NOX4 inhibition protects insulin producing cells are, however, not known. The aim of the present study was to investigate the effects of a pharmacological NOX4 inhibitor (GLX7013114) on human islet and EndoC-βH1 cell mitochondrial function, and to correlate such effects with survival in islets of different size, activity, and glucose-stimulated insulin release responsiveness. We found that maximal oxygen consumption rates, but not the rates of acidification and proton leak, were increased in islets after acute NOX4 inhibition. In EndoC-βH1 cells, NOX4 inhibition increased the mitochondrial membrane potential, as estimated by JC-1 fluorescence; mitochondrial reactive oxygen species (ROS) production, as estimated by MitoSOX fluorescence; and the ATP/ADP ratio, as assessed by a bioluminescent assay. Moreover, the insulin release from EndoC-βH1 cells at a high glucose concentration increased with NOX4 inhibition. These findings were paralleled by NOX4 inhibition-induced protection against human islet cell death when challenged with high glucose and sodium palmitate. The NOX4 inhibitor protected equally well islets of different size, activity, and glucose responsiveness. We conclude that pharmacological alleviation of NOX4-induced inhibition of beta-cell mitochondria leads to increased, and not decreased, mitochondrial ROS, and this was associated with protection against cell death occurring in different types of heterogeneous islets. Thus, NOX4 inhibition or modulation may be a therapeutic strategy in type 2 diabetes that targets all types of islets.

## 1. Introduction

The NADPH oxidases (NOX) are enzymes that produce reactive oxygen species (ROS). Seven different isoforms of the NOX family have been identified, NOX1 to NOX5, as well as dual oxidase 1 (DUOX1) and DUOX2, each with similar but distinct structural and functional characteristics [1]. NOX4 is considered to be expressed in most cell types and regulated by altered gene expression, hypoxia, and the polymerase (DNA-directed) delta-interacting protein 2 [2,3,4,5]. At physiological conditions, NOX4, by producing hydrogen peroxide and superoxide, affects cell signaling events such as those promoted by receptor tyrosine kinases [6]. In this manner, NOX4 contributes to various normal cellular functions, and it has been reported that NOX4, in myofibroblasts and endothelial cells, participates in processes such as differentiation, cytoskeletal reorganization, and transcription [7,8,9]. Other cellular processes that seem to be affected by NOX4 are epithelial to mesenchymal transition [10], ER release of Ca^2+^ [10], and apoptosis, and in the latter case, both protection and promotion thereof [11].

Lately, there has been increased interest in the interaction between NOX4 and mitochondria. NOX4 appears, at least in some cases, to localize to mitochondria [12], where it seems to function as a sensor of mitochondrial ATP with the purpose to regulate flux through the glycolytic pathway in tumor cells [13]. NOX4 may also augment mitochondrial ROS production and decrease mitochondrial function [14], possibly by inhibition of respiratory complex I [15]. In addition, it has been proposed that NOX4 inhibits mitochondrial function by downregulating Nrf2-mediated TFAM mRNA transcription, an event necessary for mitochondrial biogenesis and bioenergetics [16]. Thus, besides simply promoting deleterious levels of ROS, NOX4 may be an important modulator of mitochondrial function [14]. It has been proposed that alterations in the mitochondria of insulin producing beta-cells could be critical to the pathogenesis of type 2 diabetes [17].

We have previously demonstrated that selective NOX4 inhibition protects human islets from cytokine and palmitate + high-glucose-induced death in vitro [18] and counteracts high-fat-diet-induced glucose intolerance in mice [19]. However, the mechanisms of action underlying these effects are unknown. As NOX4 may act on mitochondria, the first aim of this study was to determine whether NOX4 inhibition affects mitochondrial function in beta-cells. Secondly, we aimed to assess differences in NOX4 inhibition-induced effect between islets depending on their functional characteristics. Human pancreatic islets have been demonstrated to be vastly heterogeneous regarding size, function, maturity, and granularity [20,21,22]. With some islet populations hypothesized to be more sensitive to gluco-lipotoxicity, it would make them critical in the development of type 2 diabetes and preferential targets in pharmacological attempts to this disease.

## 2. Materials and Methods

### 2.1. Materials

The NOX4 inhibitor GLX7013114 was kindly provided by Glucox Biotech AB (Stockholm, Sweden). GLX7013114 was characterized in a previous publication [18]. Antimycin, Oligomycin, and FCCP were purchased from Sigma-Aldrich (Darmstadt, Germany). JC-1 and MitoSOX were from Invitrogen Molecular Probes (Waltham, MA, USA).

### 2.2. EndoC-βH1 Cell and Human Islet Culture

Human EndoC-βH1 cells were cultured in ECM/fibronectin-coated plates as previously described [23,24]. Human pancreatic islets were kindly provided by the Nordic Network for Clinical Islet Transplantation. Ethical permission for work with human islets was given by the local ethics committee (Regionala etikprövningsnämnden, Uppsala, Sweden) in Uppsala, Sweden (date of approval 9 August 2017, project code Dnr 2017-283). After isolation, the islets were cultured free-floating in Sterilin dishes in CMRL 1066 medium containing 5.6 mM glucose, 10% fetal calf serum, and 2 mM L-glutamine for 1–5 days. All cultures were kept at 37 °C in a humidified atmosphere with 5% CO_2_. Information about the human islet donors is given in Supplementary Table S1.

### 2.3. Oxygen Consumption and Extracellular Acidification Rates

Oxygen consumption rates (OCRs) were determined by Seahorse Extracellular Flux Analyzer XFe96 (Seahorse Bioscience, Billerica, MA, USA). Human islets, in groups of 10, were preincubated for 3 h at 20 mM glucose before the start of analysis. After 20 min of recording at basal conditions, GLX7013114 (0.5 and 2 μM) was injected into some of the wells. After another 20 min, Oligomycin, FCCP, and Antimycin + rotenone were consecutively injected at 20–30 min intervals.

### 2.4. Mitochondrial Membrane Potential

EndoC-βH1 cells were incubated at 5.5 mM or 22.2 mM glucose in DMEM/F12 (50/50) medium and stained with 4 μM JC-1 and varying concentrations of GLX7013114 (0.1–2.0 μM) for 20 min. Cells were then washed with warm Hank’s balanced salt solution, trypsinized for 5 min, and analyzed with flow cytometry in a Becton Dickinson FACSCalibur. FL2/FL1 signal ratios were used to calculate changes in mitochondrial membrane potential.

### 2.5. Mitochondrial ROS

Mitochondrial superoxide production was measured using the MitoSox Red fluorescent probe. EndoC-βH1 cells were incubated with or without 1 μM GLX7013114 for 1 h; 5 μM MitoSox Red was added 40 min prior to analysis. Some of the cells were additionally treated with palmitate (1.5 mM), high glucose (22 mM), a combination of the two, antimycin (10 μM), and cytokines (IL-1β (20 ng/mL) + IFN-γ (20 ng/mL)) during the same 1 h incubation period. After trypsinization and washing, fluorescence intensity (580 nm) was quantified by flow cytometry.

### 2.6. ATP and ADP

A bioluminescent ADP/ATP assay kit (Abcam, ab65313) was used to examine changes in ADP and ATP levels in EndoC-βH1 cells following incubation for 20 min with 0.1–2.0 μM GLX7013114 at 5.6 mM glucose. FCCP (50 μM) was used as a positive control.

### 2.7. EndoC-βH1 Insulin Release

EndoC-βH1 cells were acclimatized for 1 h in 1.67 mM KRBH (with 0.1% BSA) and subsequently cultured in low (1.67 mM) or high glucose (17 mM) containing KRBH with 0.1–1.0 μM GLX7013114 for 1 h. The buffer was collected and insulin levels were analyzed using an insulin ELISA (Mercodia).

### 2.8. Single Islet Insulin Release and Survival Assessment after Gluco-Lipotoxic Stress

A total of 131 size-matched islets were hand-picked from three separate deceased human donors. Following 20 min of acclimatization at 2 mM glucose, each islet was incubated for 20 min in 2, 5, and 12 mM glucose in HEPES-balanced Krebs–Ringer bicarbonate buffer (KRBH) buffer with 1% bovine serum albumin at 37 °C. Buffer samples were saved for insulin analysis. The islets were subsequently cultured in CMRL1066 medium with 20 mM glucose + 0.75 mM palmitate (1% BSA). Half of the islets from each donor were supplemented with 1 μM NOX4 inhibitor (GLX7013114) to their culture medium. After 48 h, the islets were stained with bisbenzimide (10 μg/mL) and propidium iodide (PI, 10 μg/mL) for 20 min. After washing in PBS, the islets were photographed using an inverted fluorescence microscope, and the intensity of red (PI) and blue (bisbenzimide) was quantified using Image J software (National Institutes of Health, USA). Viability was assessed as the PI-to-bisbenzimide signal ratio. Samples from the graded single islet insulin release were analyzed for insulin content using ultrasensitive insulin ELISA (Mercodia); quota between the insulin release at 12 mM/5 mM and 5 mM/2 mM were calculated and, based on these results, each islet was stratified according to their glucose sensitivity. Islets with no insulin release at any glucose concentration, as well as islets missing at the time of staining, were excluded from the results.

### 2.9. Statistical Analysis

Prism 9.0 for Macintosh, Graphpad Software (San Diego, CA, USA), LLC, was used for all statistical computations. The results are given as means ± SEM. For multiple comparisons, repeated measurements one- and two-way ANOVA followed by Holm–Sidak post hoc test was used.

## 3. Results

### 3.1. GLX7013114-Mediated NOX4 Inhibition Increased Maximal Oxygen Consumption Rates in Human Islets

**Figure 1:**
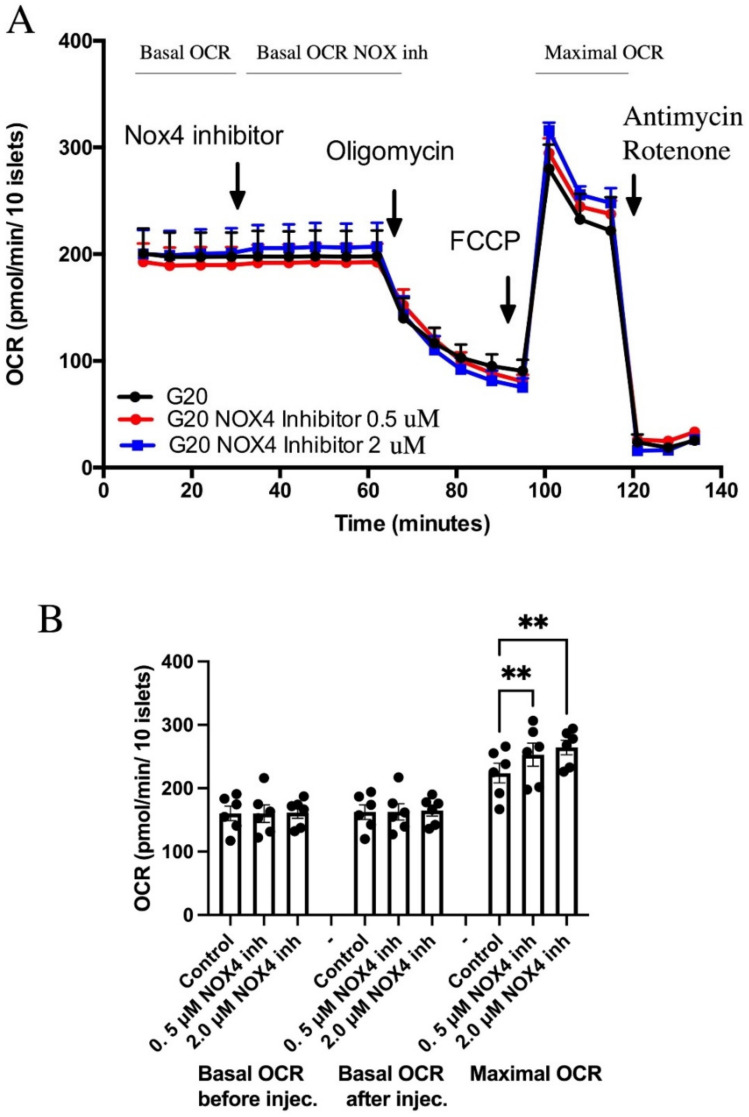
NOX4 inhibition using GLX7013114 results in increased maximal oxygen consumption rates (OCRs). Human islets were pre-incubated for 3 h in 20 mM glucose before the start of measurement. (**A**) Typical OCR recordings. (**B**) Mean ± SEM for six independent observations, each with 3–6 replicates per group. ** denotes *p* < 0.01.

To examine if inhibition of NOX4 affects human islet mitochondrial respiration, oxygen consumption rates were analyzed using the SeaHorse assay. Acute addition of GLX7013114 to human islets incubated at 20 mM glucose did not affect basal oxygen consumption rates (Figure 1). However, when challenged with the uncoupler FCCP, which maximizes respiration, GLX7013114 dose-dependently increased respiration, suggesting that NOX4 limits the respiratory chain activity when maximally stimulated. GLX7013114 affected neither proton leak nor acidification rates (Supplementary Figure S1).

### 3.2. GLX7013114 Increased the Mitochondrial Membrane Potential in EndoC-βH1 Cells

**Figure 2:**
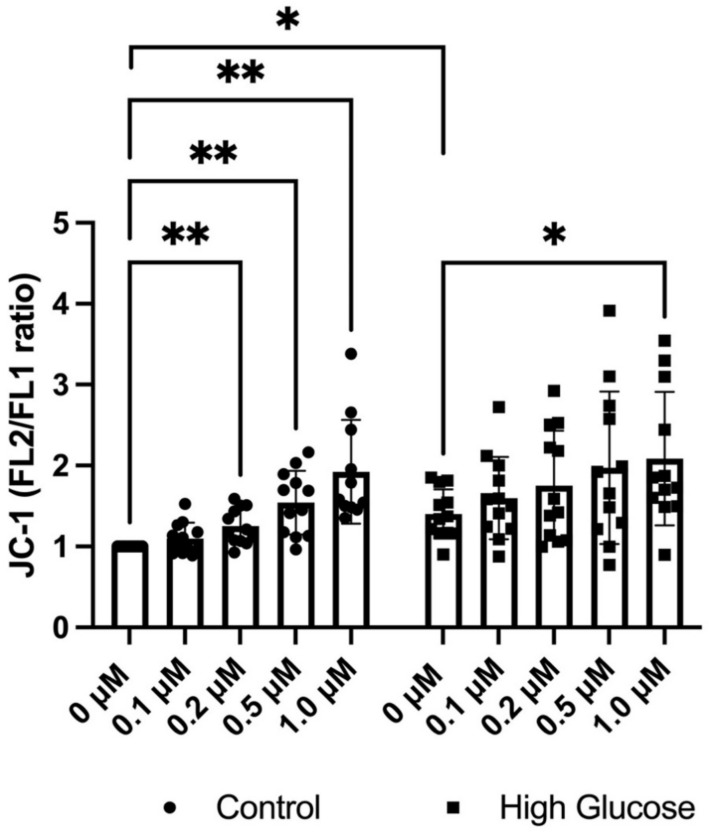
GLX7013114 increases mitochondrial JC-1 fluorescence in EndoC-βH1. EndoC-βH1 cells were labeled with 5 μM JC-1 for 20 min in the presence of different GLX7013114 concentrations. JC-1 fluorescence was detected by flow cytometry and was expressed as the FL2/FL1 ratio. High glucose alone significantly increases mitochondrial activity, with GLX7013114 potentiating this effect. The results are means ± SEM for six independent observations. * denotes *p* < 0.05 and ** denotes *p* < 0.01.

Next, we assessed the mitochondrial inner membrane potential of EndoC-βH1 cells using the fluorescent probe JC-1, which gives an increased FL_2_ (J-aggregate) signal in highly polarized mitochondria. GLX7013114 was observed to increase the mitochondrial membrane potential in a dose-dependent manner starting at 0.2 μM (Figure 2). High glucose treatment alone also increased the J-aggregate signal, with GLX7013114 potentiating this effect (Figure 2).

### 3.3. GLX7013114 Increased Mitochondrial ROS Production in EndoC-βH1 Cells

**Figure 3:**
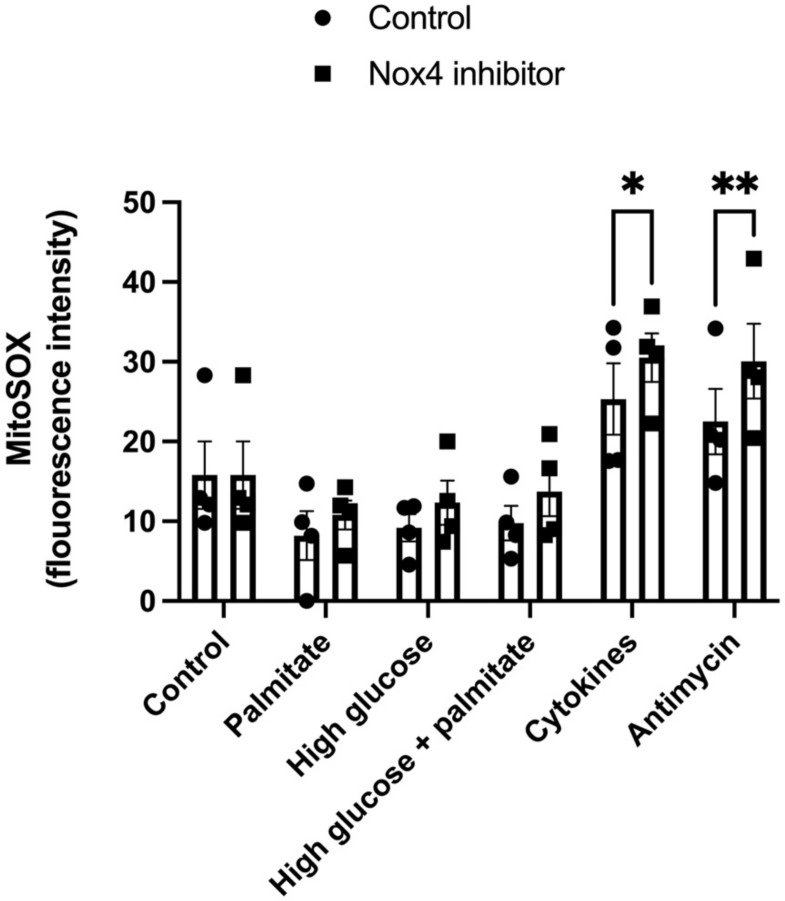
NOX4 inhibition results in increased MitoSOX fluorescence in EndoC-βH1 cells. Cells were exposed to Palmitate (1.5 mM), high glucose (22 mM), IL-1β (20 ng/mL) + IFN-γ (20 ng/mL), and Antimycin (10 μM) with and without 1 μM GLX7013114 for 1 h. During the last 40 min, the cells were labeled with 5 μM MitoSOX. Fluorescence was detected using flow cytometry. The results are means ± SEM for four independent experiments. * denotes *p* < 0.05 and ** denotes *p* < 0.01.

Using MitoSox Red as a marker for mitochondrial ROS production, we investigated if the increase in mitochondrial activity associated with NOX4 inhibition was associated with an altered mitochondrial ROS production, both at basal conditions and when exposed to beta-cell stress. Treatment with 1 μM GLX7013114 for 1 h tended to increase the MitoSox signal in the groups exposed to metabolic stressors high glucose and palmitate (Figure 3). In the presence of cytokines and Antimycin, a positive control for increased mitochondrial ROS, NOX4 inhibition resulted in an increased MitoSOX signal (Figure 3).

### 3.4. GLX7013114 Increased EndoC-βH1 Cell ATP and ATP/ADP

**Figure 4:**
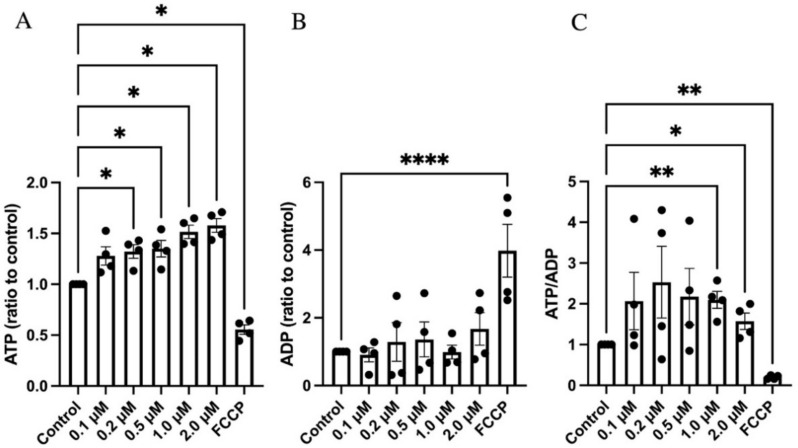
GLX7013114 increased ATP levels in EndoC-βH1 cells. EndoC-βH1 cells were exposed to different concentrations of GLX7013114 for 20 min and then analyzed for ATP (**A**) and ADP (**B**). In (**C**), ATP/ADP ratios are shown. The results are means ± SEM for four independent observations. *, **, and **** denote *p* < 0.05, *p* < 0.01, and *p* < 0.0001, respectively.

As GLX7013114 increased maximal respiration and the mitochondrial membrane potential, we next investigated whether this results in an increased ATP/ADP ratio in the beta-cells. Using a bioluminescent ADP/ATP assay, we observed that NOX4 inhibition in EndoC-βH1 cells incubated at 5.6 mM glucose increased ATP levels (Figure 4A). There was no significant change in ADP levels (Figure 4B). ATP/ADP ratios were increased at 1 μM and 2 μM of GLX7013114 (Figure 4C). The positive control for reduced ATP/ADP ratios, FCCP, promoted a clear reduction in ATP levels and a corresponding increase in ADP (Figure 4).

### 3.5. GLX7013114 Increased EndoC-βH1 Cell Glucose-Stimulated Insulin Release

**Figure 5:**
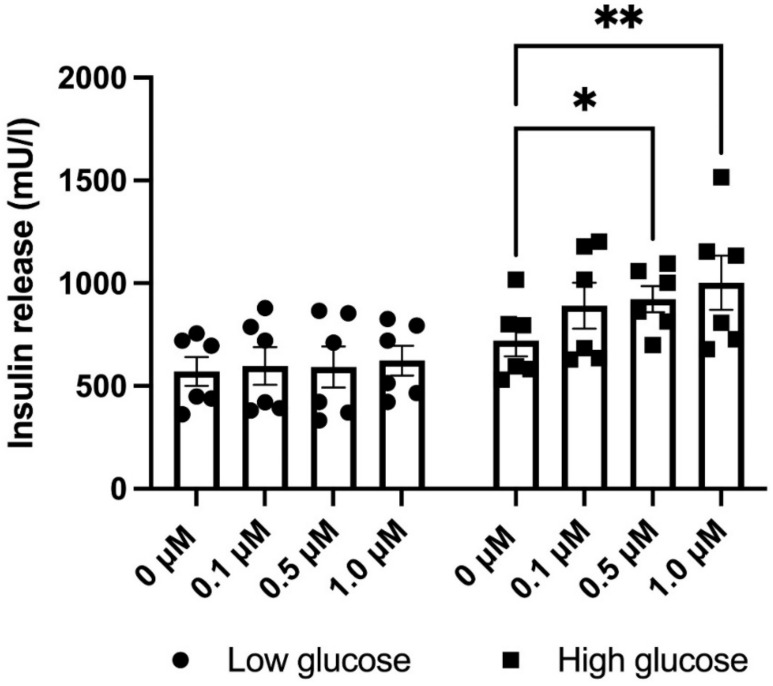
GLX7013114 increases insulin release in EndoC-βH1 cells at high glucose (17 mM), but not at low glucose (1.7 mM). Cells were pre-incubated for 1 h at a low glucose concentration (1.7 mM) and then incubated for another 60 min at 1.7 or 17 mM glucose. GLX7013114 (1 μM) was present during the last 1 h incubation period. The results are means ± SEM for six independent experiments. * and ** denote *p* < 0.05 and 0.01, respectively.

To further examine if the above-mentioned effects of NOX4 inhibition translate to alterations in insulin release, EndoC-βH1 cells were treated with varying concentrations of GLX7013114 in low (1.7 mM) or high glucose (17 mM) Krebs–Ringer bicarbonate buffer for 1 h—after which insulin levels were analyzed using an insulin ELISA. We found that NOX4 inhibition increased insulin release at high glucose (Figure 5), while insulin release at low glucose was unaffected.

### 3.6. GLX7013114 Protected Human Islets against High Glucose + Palmitate-Induced Cell Death Independently of Size, Activity, and Glucose Responsiveness

**Figure 6:**
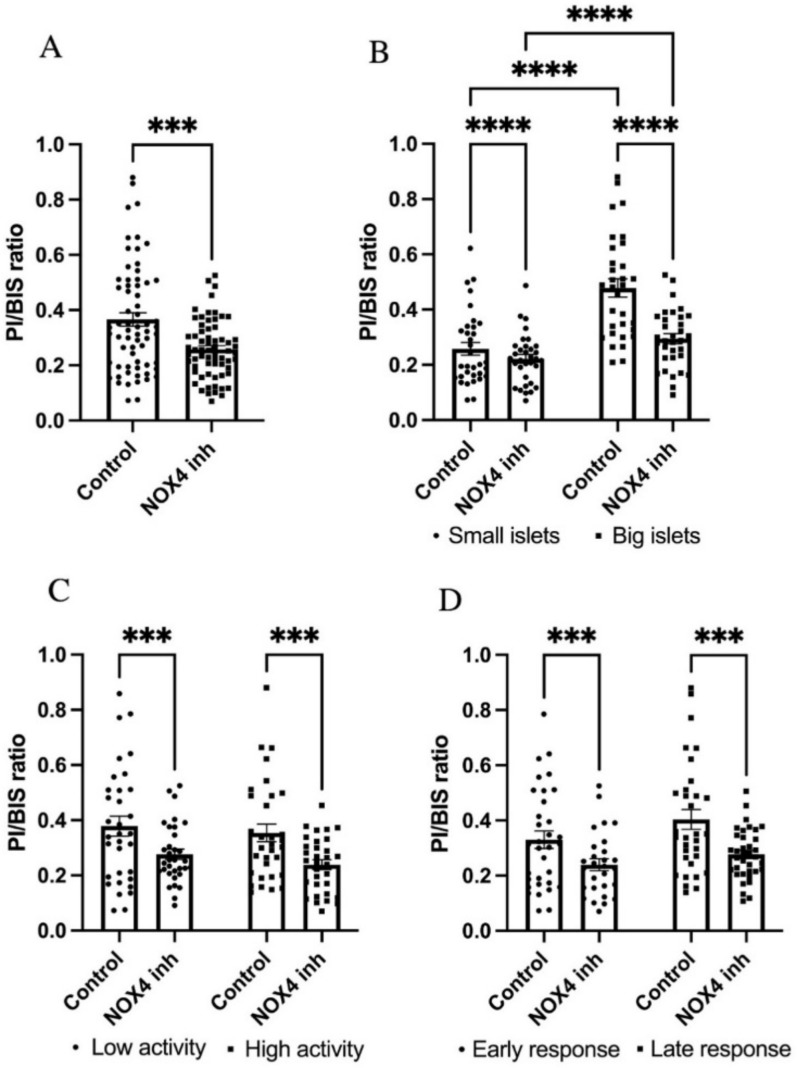
GLX7013114 protects against human islet cell death in all types of islets. Effect of 1 μM GLX7013114 on palmitate + high glucose-induced cell death in all islets is shown in (**A**). Islets were treated for 48 h and a total of 131 islets were analyzed. Prior to the treatment period, the islet insulin release at 2/5/12 mM glucose was determined. Cell death in large and small islets, with or without NOX4 inhibition, is shown in (**B**). NOX4 inhibition resulted in a significant protection of both highly active islets and islets with low activity (**C**). GLX7013114 significantly reduced the PI/BIS ratio in both early response islets and late response islets (**D**). A total of 144 islets from three different organ donors were analyzed. The results are mean ± SEM. *** and **** denote *p* < 0.001 and *p* < 0.0001, respectively.

To examine the effect of NOX4 in different islet sub-populations in vitro, we studied the insulin release of single islets and then the survival of the same islets following exposure to gluco-lipotoxic conditions with or without NOX4 inhibition. Insulin release was measured at 2, 5, and 12 mM glucose (30 min at each concentration). Insulin levels at 12 mM were compared with 5 mM, and those at 5 mM were compared with 2 mM for each islet, and ratios between the levels were calculated to stratify the islets according to their glucose response. Out of the 144 included islets, 13 were excluded owing to no insulin secretion or were missing at the time of staining (6 treated islets, 7 control islets). We first observed that the total islet population receiving GLX7013114 treatment had significantly lower levels of cell death compared with the control (Figure 6A). The islets were then evenly subcategorized as large or small based on the BIS-signal strength, as well as low/highly active and early-/late-responding based on their insulin release ratios at 12/5 mM and 5/2 mM glucose. Large islets were more susceptible to the damaging effects of gluco-lipotoxicity compared with small islets (Figure 6B), while no significant differences were observed between islets with high and low functional activity. GLX7013114 significantly reduced cell death in both small and large islets (Figure 6B), low and high activity islets (Figure 6C), and early and late response islets (Figure 6D).

## 4. Discussion

GLX7013114 is a highly selective NOX4 inhibitor (IC_50_ = 0.3 μM) that counteracts human islet cell death in vitro [18]. Here, we report that NOX4 inhibition using this inhibitor modulates mitochondrial activity in human islets by acutely increasing maximal OCR, the mitochondrial inner membrane potential, and ATP levels. Somewhat surprisingly, while NOX4 is an ROS producing enzyme, inhibition of it using GLX7013114 increased mitochondrial ROS in beta-cells exposed to proinflammatory cytokines and Antimycin, which is opposite to what has previously been reported [15]. It may be that the amount of ROS produced by NOX4 in beta-cell mitochondria is insignificant in comparison with the amounts produced by the respiratory chain. In that case, it is likely that increased mitochondrial ROS production is explained by enhanced mitochondrial activity, as this would be coupled with an increased electron leakage, and thus augmented mitochondrial ROS production. This suggests that ROS production by NOX4 in mitochondria is not enough to promote oxidative stress, and that it instead controls key control events in the bioenergetics of mitochondria. Indeed, the effects we observed on respiration rates were acute and present at a high glucose concentration, supporting this regulatory role of NOX4 under conditions of ample supply of nutrients. Moreover, our insulin release results in GLX7013114-treated EndoC-βH1 cells were in line with this, as NOX4 inhibition potentiated insulin release in high glucose conditions, but not at low glucose. This scenario is supported by the finding that NOX4 targets specifically respiratory complex I in endothelial cells, resulting in reduced respiration [14].

NOX4 has previously been suggested to function as a mitochondrial energy sensor and glycolytic regulator in tumor cells [13]. This, however, does not seem to be the case in human islet cells as we did not observe any signs of altered glycolysis (acidification rates) in response to GLX7013114 in our Seahorse experiments.

Our previous findings on GLX351322, a NOX1-, 4-, and 5-inhibitor, yielded contradictory results regarding insulin release, inhibiting human islet insulin release in response to glucose and palmitate [19]. This demonstrates the intricate roles of the different NOX-isoforms, but suggests that NOX4 in particular has important regulatory effects on beta-cell bioenergetics. It is conceivable that the metabolic conditions present in T2D dysregulates NOX4 into a hyperactive state that subsequently results in late-stage impairment of beta-cell mitochondrial function, as previously discussed [17]. On the other hand, we have previously demonstrated that NOX4 inhibition decreases total ROS levels in beta-cells [18]. Therefore, the protective effects of NOX4 inhibition we observed on stressed beta-cells could stem from both specific effects on cellular energy balance, as well as a general decrease in ROS.

Heterogeneity between islets has been established [20,21,22]. It can be speculated that highly active islets, and islets that respond at higher glucose concentrations, are more prone to the damaging effects of gluco-lipotoxicity, as impaired glucose tolerance usually presents prior to onset of type 2 diabetes. Regional differences in beta-cell loss in the pancreas of diabetic patients, as well as findings that the most functional islets are more prone to develop amyloid deposits, may support this [25,26]. However, by measuring the insulin release of single islets from three separate human donors, we were able to classify each islets’ glucose sensitivity and activity prior to exerting them to gluco-lipotoxicity, and observed that NOX4 inhibition protected islets regardless of their size, insulin response characteristics, or activity. Thus, NOX4-induced beta-cell death seems to occur in all types of islets and may involve a mechanism that acts independently of the insulin releasing activity of the islet.

While our findings support the notion that NOX4 is harmful in stressed human beta-cells, it is also possible that short-term NOX 4 activation mediates beneficial effects. Mitochondrial overactivation and metabolic maladaptation have recently been suggested to be an early response to chronic glucose stress in beta cells [27], and protecting mitochondria from this nutrient overload may preserve normal beta-cell function [28]. NOX4 inhibition might modulate the mitochondrial activity of beta-cells enough for them to overcome the stress from a diabetic milieu. Further research regarding NOX4 function in beta-cell mitochondria during prolonged glucose-induced stress is warranted.

## 5. Conclusions

We conclude that NOX4 plays a role in beta-cell mitochondrial activity regulation, with NOX4 inhibition or modulation improving their function and survival in vitro under conditions of short-term stress. We are currently conducting in vivo experiments with selective NOX4 inhibitors in diabetic humanized mice, in order to establish if these findings translate into in vivo models and longer time periods. It is possible that intermittent and partial NOX4 inhibition, for example, after high-fat feeding, might sufficiently reduce metabolic stress.

## Data Availability

The data presented in this study are available on request from the corresponding author.

## Supplementary Materials

The following are available online at https://www.mdpi.com/article/10.3390/biomedicines9121865/s1. Supplementary Figure S1: NOX4 inhibition does not affect human islet proton leak (upper panel) or human islet extracellular acidification rate (ECAR)(lower panel). Human islets were analyzed as given in Figure 1. Supplementary Table S1: Information on human islet organ donors used for human islet isolation.
