# Supplementary material for: Pharmacological Inhibition of NOX4 Improves Mitochondrial Function and Survival in Human Beta-Cells
Source: Biomedicines. 2021 Dec 8;9(12):1865. doi: 10.3390/biomedicines9121865 (PMC8698703; doi:10.3390/biomedicines9121865)
Supplement: Supplementary file 1 [file biomedicines-09-01865-s001.zip › biomedicines-1419758-supplementary-done.pdf]

biomedicines

MDPI

*Article*

# Pharmacological Inhibition of NOX4 Improves Mitochondrial Function and Survival in Human Beta-Cells

**Andris Elksnis [1], Jing Cen [1], Per Wikström [2], Per-Ola Carlsson [1,3] and Nils Welsh [1,*]**

[1] Science for Life Laboratory, Department of Medical Cell Biology, Uppsala University, Box 571, SE-751 23 Uppsala, Sweden; andris.elksnis@mcb.uu.se (A.E.); jing.cen@mcb.uu.se (J.C.); Per-Ola.Carlsson@mcb.uu.se (P.-O.C.)
[2] Glucox Biotech AB, Frälsegårdsvägen 8, SE-179 97 Färentuna, Sweden; per.wikstrom@glucoxbiotech.com
[3] Department of Medical Sciences, Uppsala University, SE-751 85 Uppsala, Sweden
* Correspondence: nils.welsh@mcb.uu.se; Tel.: +46-184-714-212

**Supplemental Table S1.** Information on human islet organ donors used for human islet isolation.

| **Islet preparation** | **1** | **2** | **3** | **4** | **5** | **6** |
|---|---|---|---|---|---|---|
| Unique identifier | H2491 | H2494 | H2496 | H2512 | H2516 | H2517 |
| Donor age (years) | 71 | 81 | 73 | 56 | 55 | 51 |
| Donor sex (M/F) | M | F | M | F | M | F |
| Donor BMI ($kg/m^2$) | 22,4 | 21,1 | 23,2 | 24,6 | 22,4 | 28,1 |
| Donor $HbA_{1c}$ (mmol/mol) | 41 | 34 | 38 | 40 | 36 | 35 |
| Origin/source of islets | ECIT | ECIT | ECIT | ECIT | ECIT | ECIT |
| Islet isolation centre | Uppsala | Uppsala | Uppsala | Uppsala | Uppsala | Uppsala |
| Donor history of diabetes? Please select yes/no from drop down list | No | No | No | No | No | No |
| Donor cause of death | Not known | Not known | Not known | Not known | Not known | Not known |
| Warm ischaemia time (h) | Not known | Not known | Not known | Not known | Not known | Not known |
| Cold ischaemia time (h) | 21:52 | Not known | 13:35 | 21:54 | 8:57 | 11:37 |
| Estimated purity (%) | Not determined | 69 | 98 | 95 | 77 | 69 |
| Estimated viability (%) | Not determined | Not determined | Not determined | Not determined | Not determined | Not determined |
| Total culture time | All less than 5 days | All less than 5 days | All less than 5 days | All less than 5 days | All less than 5 days | All less than 5 days |
| Glucose-stimulated insulin secretion | 2,2 | 8,9 | 4,4 | 7,9 | 10,0 | 17,7 |

| | | | | | | |
|---|---|---|---|---|---|---|
| (ratio 17 mM/1.7 mM glucose) | | | | | | |
| Handpicked to purity? | Yes | Yes | Yes | Yes | Yes | Yes |

ECIT stands for European Consortium for Islet Transplantation (https://ecit.dri-sanraffaele.org).

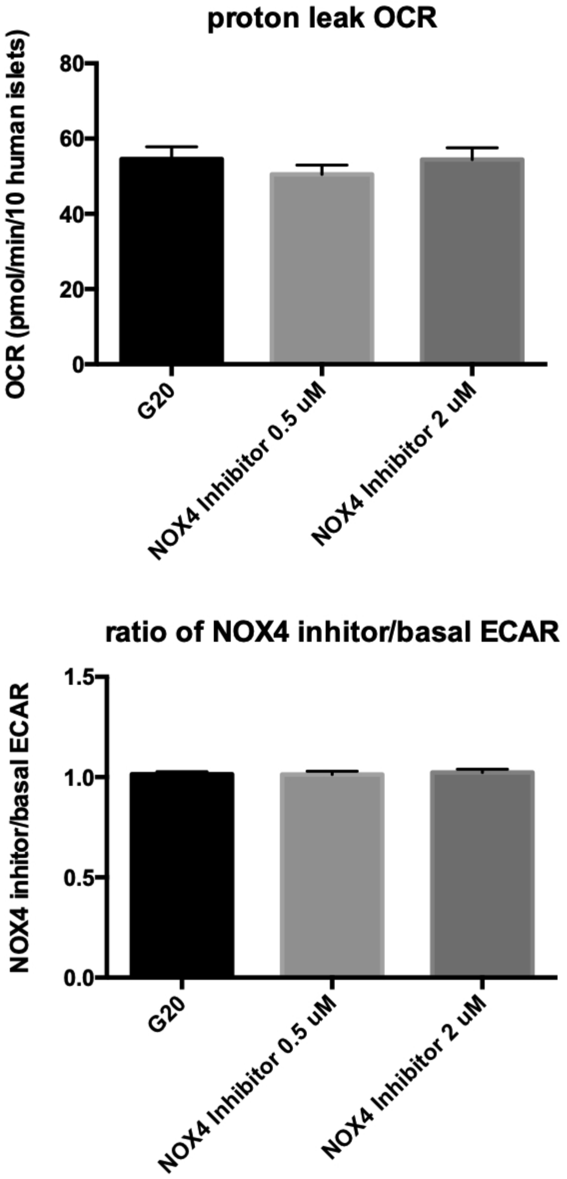


**Supplemental Figure S1.** NOX4 inhibition does not affect human islet proton leak (upper panel) or human islet extracellular acidification rate (ECAR)(lower panel). Human islets were analyzed as given in Figure 1.
